# A 27-Year Experience with Atrioventricular Septal Defect Correction

**DOI:** 10.1055/a-2536-8640

**Published:** 2025-03-28

**Authors:** Mina Farag, Mireia Isern Hacker, Philippe Grieshaber, Elizabeth Fonseca Escalante, Matthias Karck, Raoul Arnold, Matthias Gorenflo, Tsvetomir Loukanov

**Affiliations:** 1 Department of Cardiac Surgery, Division of Congenital Cardiac Surgery, Heidelberg University, Congenital, Heidelberg, Germany; 2 Department of Paediatric Cardiology, Heidelberg University, Congenital, Heidelberg, Germany

**Keywords:** cardiac, congenital heart disease, heart valve surgery, outcomes (mortality, morbidity), reoperation

## Abstract

**Background:**

This single-center study investigated long-term outcomes after surgical correction of atrioventricular septal defect (AVSD).

**Methods:**

A total of 248 patients underwent biventricular repair for AVSD between 1995 and 2022. A total of 208 (83.9%) patients had complete (cAVSD), 29 (11.7%) partial (pAVSD), and 11 (4.4%) transitional AVSD (tAVSD). Associated cardiovascular anomalies were present in 88 (35.5%) cases and 61 (24.6%) patients were born prematurely. Median age at repair was 7.1 for cAVSD, 23.7 for pAVSD, and 13 months for tAVSD.

**Results:**

Overall survival or reoperation incidence did not differ significantly between AVSD types and improved significantly over surgical eras. Survival of the entire cohort was 88.3% at 10, 83.8% at 15, and 79.6% at 25 years. Prematurity (hazard ratio [HR]: 2.43, *p* = 0.029), low weight (<4 kg) (HR: 3.05, *p* = 0.028), and partial cleft closure (HR: 2.43, *p* = 0.037) were independent risk factors for mortality. Forty-eight patients (19.4%) underwent a total of 64 reoperations over the study period. The main indication for reoperation was left atrioventricular valve regurgitation (LAVVR) with 55/64 procedures. However, 36% of procedures were performed to address several lesions, with left ventricular outflow tract obstruction being the second most common indication. Freedom from reoperation was 78.2, 75.8, and 72.5% at 10, 15, and 25 years, respectively. The incidence of reoperation increased significantly in association with early postoperative LAVVR ≥ I–II° (HR: 2.6, 95% confidence interval [CI]: 1.4–4.7, *p* = 0.002) and presence of residual cardiac defects (HR: 2.0, 95% CI: 1.1–3.6, *p* = 0.018).

**Conclusion:**

While LAVVR is the main indication for reoperation, a significant proportion of procedures address additional pathologies. Premature patients and those with associated cardiovascular anomalies should receive special attention during postoperative follow-up.

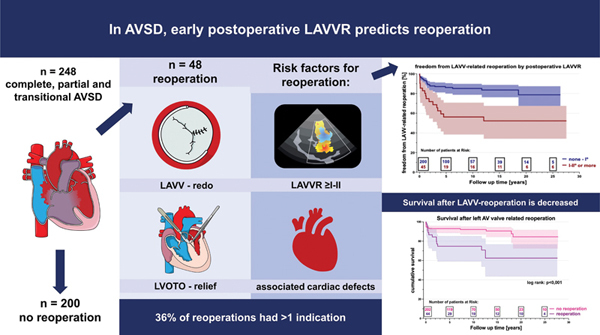

## Introduction

Atrioventricular septal defects (AVSD) result from disturbed endocardial cushion development, causing various degrees of septal malformations at atrial and ventricular level. 1 While the complete form (cAVSD) requires intervention in early infancy due to the hemodynamically important ventricular septal defect (VSD) component, treatment for transitional (tAVSD) and partial (pAVSD) AVSD can be planned according to patients' clinical status. Due to improved early as well as late survival, the number of patients becoming candidates for reintervention is steadily increasing. Several studies have reported that severe left atrioventricular valve regurgitation (LAVVR) is the primary indication for repeated surgical correction, with incidences between 5 and 19% during long-term follow-up. 2 3 However, the impact of reinterventions for associated lesions such as left ventricular outflow tract obstruction, residual shunts, or surgeries related to associated cardiac anomalies and their influence on risk profile of these patients is less often characterized. Reoperations for LAVVR pose a considerable challenge and put a significant burden on affected patients, whereas valve replacement is still associated with considerable mortality and morbidity, especially in younger patients. 4 The benefits of early repair and the management of associated cardiac malformations are still controversial. 5 The aim of this study was to identify potential risk factors associated with mortality and reoperation after surgical correction of AVSD at a single institution over a 27-year period.

## Methods

Between January 1995 and December 2022, a total of 248 patients underwent AVSD repair at this institution's hospital. Of those, 208 (83.9%) had complete, 29 (11.7%) had partial, and 11 (4.4%) had transitional AVSD. Patients with unbalanced ventricles that did not allow for biventricular repair were excluded from this study. Further five patients that met the inclusion criteria were excluded due to missing clinical data. Borderline or unbalanced ventricles were defined as nonapex-forming left ventricle ( *n* = 8) or reduced right ventricular area ( *n* = 2). The distinction between transitional AVSD and other forms was made in case of restrictive shunting over the ventricular septum. Pre-, peri-, and postoperative follow-up data were extracted retrospectively from digital patient files. For the most recent clinical status, the patients' cardiologists or pediatricians were contacted. Follow-up examinations were performed at our outpatient clinic or by referring physicians. Clinical status and last echocardiographic findings were recorded. The degree of valvular insufficiency was graded as mild (I), moderate (II), or severe (III) according to the width and depth of the insufficiency jet. Significant stenosis of the native valves was recorded if the mean gradient was equal to or exceeded 6 mm Hg. Echocardiographic findings were obtained from our clinical data bank, which is linked to the image archive. However, especially in the earlier years of the study, images were limited in their availability and only the written report was used for data collection. “Postoperative LAVVR” was defined as the first recorded postoperative LAVV function (i.e., in the intraoperative transesophageal echocardiography or within the first postoperative days). Disclosure from the residents' register was obtained for patients lost to clinical follow-up, for survival analysis. Follow-up was 95.6% complete. Mean survival follow-up time was 9.2 ± 8.1 years, whereas time to last follow-up was 8.9 ± 7.9 years (standard deviation). Over 20 years follow-up data were available for 33 patients. Survival time was calculated separately from follow-up time and was updated according to disclosure from registration office.

The need for individual informed consent was waived by our institution's ethics committee due to the retrospective nature of the study (approval number: S-775/2022). Early mortality and early reoperation were defined as events occurring within 30 days or before hospital discharge. Late pacemaker dependency was defined as occurring after hospital discharge. The study period was divided into three surgical eras; early between 1995 and 2004; recent between 2005 and 2013, and current from 2014 to 2022.

Primary endpoints of this study were overall survival and freedom from reoperation; secondary endpoints were risk factors for mortality and reoperation.

## Surgical Technique

All procedures were performed via median sternotomy and bicaval (or tricaval in case of left persisting superior vena cava [LPSVC]) cannulation. Cardioplegic arrest was achieved through cold cardioplegia (Bretschneider) infusion delivered via the aorta. Moderate hypothermia between 28 and 32°C rectal temperature, according to AVSD type, was utilized. In selected cases concomitantly addressing multiple lesions, lower rectal temperatures were targeted. Primary repair for pAVSD was achieved with a single atrial patch and cleft closure. Repair for tAVSD required the closure of the associated VSD component with pledged sutures. In patients with cAVSD, repair techniques included the two-patch technique, 6 the single-patch technique 7 as well as the modified single-patch technique. 8 9 The coronary sinus was almost always left draining to the right atrium. Cleft closure was primarily achieved with interrupted sutures between the septal edge and free margin of the bridging leaflets, as much as possible. Over the progression of surgical eras, additional valve reconstruction strategies such as annuloplasty were used. Reoperations were performed via median resternotomy and access to the LAVV was achieved through the right atrium.

## Statistical Analysis

Normally distributed continuous variables were described as mean ± standard deviation, non-normally distributed variables as median with interquartile range. Categorical variables were indicated with relative and absolute frequency and differences between groups tested with Pearson's chi-square or Fisher's exact test. Freedom from reoperation and survival were analyzed with Kaplan–Meier estimates and differences between groups tested with the log-rank test.

Cox regression analysis was conducted to define risk factors for reoperation and mortality. First, univariable Cox regression was performed for selected variables. All variables with *p* ≤ 0.200 were then included in a multivariable regression model and eliminated in a backward stepwise method if *p* > 0.100. Results were described as hazard ratio (HR) with a 95% confidence interval (CI).

Significance level for all analyses was defined a priori as *p* < 0.05. Missing values were treated as empty cells. Statistical analysis was performed with the software SPSS 27.0 (SPSS Inc, Chicago, Illinois, United States).

## Results

### Patient Characteristics

Detailed demographics are depicted in Table 1 . Median age at the time of repair was 7.9 months (interquartile range [IQR]: 5.3–17.2) and median weight was 6 kg (IQR: 4.9–8.5), with a trend toward earlier correction across the three surgical eras. In 71.6% of cAVSD patients, corrective surgery occurred within infancy, compared with 45.5% of tAVSD and 17.2% of pAVSD. Overall, 17 (6.9%) patients were younger than 4 months and 12 (4.8%) weighed less than 4 kg.

**Table 1:** Patient characteristics stratified by atrioventricular septal defect type and for total group

Patient characteristics	Complete, *n* = 208	Partial, *n* = 29	Transitional, *n* = 11	Total group, *n* = 248
Male	89 (42.8%)	11 (37.9%)	6 (54.5%)	106 (42.7%)
Premature	55 (26.4%)	4 (13.8%)	2 (18.2%)	61 (24.6%)
Age at repair (mo), median [IQR]	7.1 [5.2–13.6]	23.7 [13.8–260]	13 [6.1–25.7]	7.9 [5.3–17.2]
Age < 4 mo	14 (6.8%)	2 (6.9%)	1 (9.1%)	17 (6.9%)
Weight at repair (kg), median [IQR]	5.7 [4.9–7.5]	10.3 [8.3–45.3]	8.5 [4.7–11.3]	6.0 [4.9–8.5]
Weight < 4 kg	10 (4.8%)	1 (3.4%)	1 (9.1%)	12 (4.8%)
Complex AVSD	75 (36.1%)	9 (31%)	4 (36.4%)	88 (35.5%)
Coarctation of the aorta	14 (6.8%)	2 (6.9%)	1 (9.1%)	17 (6.9%)
Tetralogy of Fallot	8 (3.9%)	0 (0%)	0 (0%)	8 (3.2%)
RVOTO incl. ToF	19 (9.1%)	1 (3.4%)	0 (0%)	20 (8.1%)
Anomalous venous return and LPSVC	15 (7.2%)	1 (3.4%)	0 (0%)	16 (6.5%)
Borderline unbalanced ventricles	10 (4.8%)	0 (0%)	0 (0%)	10 (4.0%)
Abnormal papillary muscle	27 (13.0%)	1 (3.4%)	2 (18.2%)	30 (12.1%)
Trisomy 21	148 (71.2%)	10 (34.5%)	4 (36.4%)	162 (65.3%)
Prior pulmonary artery banding	46 (22.1%)	–	–	–
Preoperative pulmonary hypertension	152 (73.1%)	10 (34.5%)	5 (45.5%)	167 (67.3%)
Preoperative LAVVR > II	19 (9.1%)	6 (20.7%)	1 (9.1%)	26 (10.5%)

Abbreviations: AVSD, atrioventricular septal defect; IQR, interquartile range; LAVVR, left atrioventricular valve regurgitation; LPSVC, left persisting superior vena cava; RVOTO, right ventricular outflow tract obstruction; ToF, tetralogy of Fallot.

Trisomy 21 was encountered in 162 (65.3%) patients; 12 (4.8%) patients had other genetic syndromes such as Ellis–van-Creveld syndrome and Au–Kline syndrome. Premature birth (<37th gestational week) was encountered in 61 (24.6%). Complex-associated cardiovascular anomalies were present in 88 cases (35.5%). These included—among other malformations—17 (6.9%) patients with coarctation of the aorta (CoA) and 20 patients with right ventricular outflow obstruction (RVOTO) (8.1%) of whom 8 (3.2%) due to tetralogy of Fallot (ToF) association. Ten patients (4%) presented with borderline unbalanced ventricles suitable for biventricular repair. Complex AV valve configurations, such as abnormal papillary muscle anatomy (including single papillary muscle and narrow left ventricle papillary muscle) were encountered in 30 (12.1%) and double-orifice mitral valve in 8 (3.2%) cases. Systemic venous anomalies such as azygos or hemiazygos continuity and/or LPSVC were noted in 16 (6.5%) cases.

Twenty-two (8.9%) patients had undergone previous cardiac surgery other than central pulmonary artery banding (cPAB). These were PDA ligature (9/22), correction for aortic coarctation (8/22) or RVOTO relief (4/22) and one modified Blalock–Taussig shunt. In the cAVSD cohort, 46 patients (22.1%) received prior cPAB. A decrease in cPAB from 36.9 to 13.2% across surgical eras was noted. Median age at pulmonary artery banding (PAB) was 2.9 months (IQR: 0.8–5) and median duration of PAB was 10.1 months (IQR: 7.7–16.1). Complex-associated malformations were recorded in 58.7% of this group.

### Operative Data

For cAVSD correction, the double-patch technique was most frequently used (64.4% of corrections). The double-patch and the modified single-patch techniques were used with equal frequency in the current surgical era. Detailed operative and perioperative characteristics are presented in Table 2 *.* Additional valvular reconstruction strategies such as commissuroplasty and annuloplasty became more frequent (performed in 35% of patients from 2014 to 2022 vs. 16% of patients in 1995–2013).

**Table 2:** Operative characteristics and outcome stratified by atrioventricular septal defect type and for total group

Operative characteristics and outcome	Complete, *n* = 208	Partial, *n* = 29	Transitional, *n* = 11	Total group, *n* = 248
Single patch	5 (2.4%)	29 (100%)	0 (0%)	36 (14.5%)
Double patch	134 (64.4%)	0 (0%)	1 (9.1%)	135 (54.4%)
Modified single patch	69 (33.2%)	0 (0%)	10 (90.9%)	79 (31.9%)
Bypass time, mean ± SD	127 min ± 43	96 min ± 33	91 min ± 36	122 min ± 43
Aortic cross-clamp time, mean ± SD	74 min ± 26	56 min ± 18	48 min ± 22	71 min ± 26
Complete cleft closure	168 (80.8%)	28 (96.6%)	11 (100%)	207 (83.5%)
Commissure or annuloplasty	57 (27.4%)	2 (6.9%)	3 (27.3%)	62 (25.0%)
Concomitant operation	77 (37.0%)	3 (10.3%)	2 (18.2%)	82 (33.2%)
RVOTO relief	16 (7.7%)	0 (0%)	0 (0%)	16 (6.5%)
CoA repair	2 (1%)	1 (3.4%)	1 (9.1%)	4 (1.6%)
PDA ligature	40 (19.2%)	2 (6.9%)	0 (0%)	42 (17%)
Postoperative LAVVR >I–II°	41 (19.7%)	4 (13.8%)	1 (9.1%)	46 (18.5%)
ICU length of stay, median [IQR]	9 d [6–18]	4 d [3–8]	4 d [3–6]	9 d [5–16]
Hospital length of stay, median [IQR]	17 d [12–28]	13 d [10–16]	11 d [8–20]	16 d [11–25]
In-hospital reoperation	12 (5.8%)	1 (3.4%)	0 (0%)	13 (5.2%)
Late reoperation	29 (13.9%)	3 (10.3%)	3 (27.3%)	35 (14.1%)
30-d mortality	11 (5.3%)	0 (0%)	0 (0%)	11 (4.4%)
Late mortality	14 (6.7%)	2 (6.9%)	1 (9.1%)	17 (6.9%)

Abbreviations: CoA, Coarctation of the aorta; ICU, intensive care unit; IQR, interquartile range; PDA, persisting ductus arteriosus; RVOTO, right ventricular outflow tract obstruction; SD, standard deviation.

Mean bypass time was 122 minutes ± 43 and mean cross-clamp time was 71 minutes ± 26. In 16.5% of patients, partial cleft closure was performed due to marked hypoplasia of valvular tissue. Concomitant surgical correction for right ventricular outflow tract obstruction or coarctation was needed in 6.5 and 1.6% of patients, respectively.

### Mortality

**Fig. 1:**
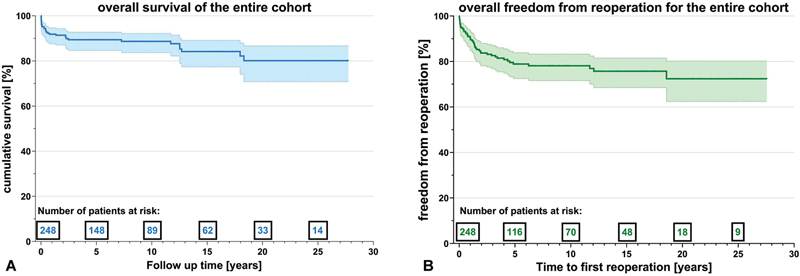
( **A** ) Left: survival estimates of the entire cohort. ( **B** ) Right: freedom from reoperation of the entire cohort.

Median follow-up duration was 9.2 ± 8.1 years. Overall survival of the entire study group was 86.7% with survival estimates being 91.8% at 1 year [95% CI: 0.88–0.95], 88.3% at 10 years [95% CI: 0.84–0.93], and 79.6% at 20 and 25 years [95% CI: 0.72–0.88] ( Fig. 1A ). A total of 33 patients were nonsurvivors.

Sixteen patients (6.5%) died before hospital discharge. The observed in-hospital mortality decreased significantly across the three surgical eras from 12.1% in the early to 1.8% in the current era ( *p* < 0.001) (30-day mortality: 4.4 and 0%, respectively). Nonsurvivors were a median 2.9 months younger and 1.5 kg lighter than patients who survived the hospital stay. Furthermore, 50% of nonsurvivors were born prematurely and 43.8% suffered a postoperative pulmonary hypertensive crisis. Additionally, incomplete cleft closure and postoperative LAVVR ≥ I–II° were encountered in seven patients, respectively. Of the entire cohort, primary chest closure was not possible in four patients, all of whom were nonsurvivors.

Late mortality was 6.9% (17/248). Cardiac failure was the most common cause of death (56.3% of early and 41.2% of late deaths), followed by pulmonary hypertension for early deaths (31.3%) and noncardiac causes for late deaths (17.7%). Ten late deaths occurred within the first 3 years after AVSD correction. Two patients died 18 years after correction from unknown causes.

Survival did not differ significantly for the different AVSD types, despite numerically lower early mortality for pAVSD compared with cAVSD (3.4 vs. 7.2%, *p* = 0.45). Of the 11 patients with tAVSD, 1 died after hospital discharge.

Associated cardiac anomalies with significantly higher mortality were systemic venous anomalies (31.3% mortality, *p* = 0.026). Survival of cAVSD patients after cPAB was numerically reduced, albeit not reaching statistical significance (78.3 vs. 88.8% overall survival, *p* = 0.169).

Univariable analysis correlated low weight (<4 kg), age (<4 months), premature birth, postoperative LAVVR ≥ I–II°, partial cleft closure, and early surgical era with mortality. Upon multivariate Cox regression analysis low weight ( *p* = 0.028), prematurity ( *p* = 0.029), partial cleft closure ( *p* = 0.037), and early surgical era ( *p* = 0.031) remained independent predictors of mortality ( Table 3 ).

**Table 3:** Risk factors for mortality and for reoperation by univariable and multivariable Cox regression analysis

Risk factors for mortality	Univariable HR	95% CI	*p* -Value	Multivariable HR	95% CI	*p* -Value
Age < 4 mo	2.80	1.09–7.31	0.033			
Weight < 4 kg	5.78	2.38–14.08	<0.001	3.05	1.13–8.28	0.028
Premature	2.49	1.22–5.04	0.012	2.43	1.10–5.40	0.029
PAB	1.75	0.83–3.73	0.144			
Trisomy 21	1.64	0.74–3.63	0.227			
Preoperative LAVVR ≥ II–III°	1.59	0.61–4.13	0.342			
Postoperative LAVV regurgitation ≥ I–II°	2.90	1.43–5.90	0.003			
Reoperation	2.83	1.42–5.66	0.003			
Partial cleft closure	2.81	1.28–6.13	0.010	2.44	1.06–5.61	0.037
Complex AVSD	1.55	0.78–3.09	0.208			
Early surgical era	4.46	1.60–12.44	0.004	3.62	1.13–11.62	0.031
**Risk factors for reoperation**	**Univariable HR**	**95% CI**	***p*** **-Value**	**Multivariable HR**	**95% CI**	***p*** **-Value**
Age < 4 mo	1.52	0.55–4.23	0.424			
Weight < 4 kg	1.33	0.32–5.48	0.695			
Trisomy 21	0.89	0.50–1.60	0.701			
Preoperative LAVV regurgitation ≥ II–III°	2.07	1.00–4.29	0.049			
Postoperative LAVV regurgitation ≥ I–II°	2.93	1.62–5.32	<0.001	2.58	1.41–4.73	0.002
Partial cleft closure	1.78	0.88–3.75	0.108			
Complex AVSD	2.21	1.25–3.90	0.007	2.02	1.13–3.62	0.018
Early surgical era	1.51	0.38–1.74	0.228			

Abbreviations: AVSD, atrioventricular septal defect; CI, confidence Interval; HR, hazard ratio; LAVV, left atrioventricular valve; PAB, pulmonary artery banding.

### Reoperations

A total of 48 (19.4%) patients required 64 reoperations over the entire follow-up period. Freedom from reoperation was 90.5% [95% CI: 0.87–0.95] after 1 year, 78.2% [95% CI: 0.73–84], and 72.5% [95% CI: 0.64–0.81] at 10 and 25 years, respectively ( Fig. 1B *)* . Twelve patients required a second reoperation and two patients a third reoperation.

**Fig. 2:**
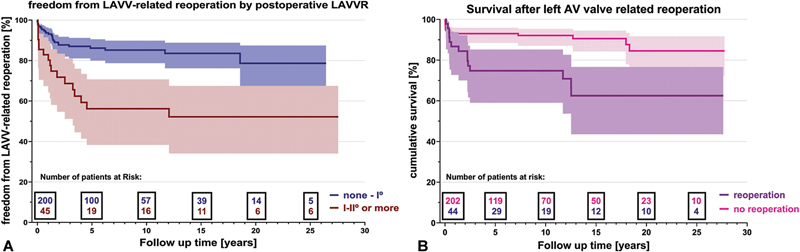
( **A** ) Left: Kaplan–Meier estimate for freedom from LAVV-related reoperation stratified by degree of early LAVV regurgitation: none–I° versus I–II° or more. ( **B** ) Right: Kaplan–Meier estimate for survival of patients with LAVV-related reoperation compared with no LAVV reoperation. LAVV, left atrioventricular valve.

The median interval from initial AVSD correction to first reoperation was 1.1 years (IQR: 0.2–2.3) with the median age at first reoperation being 1.8 years (IQR: 1.1–3.3). The need for reoperation did not differ significantly based on AVSD type ( *p* = 0.37). The severity of early postoperative LAVVR significantly correlated with higher reoperation rate ( *p* < 0.001) ( Fig. 2A ). Patients with partial cleft closure (41/248) had significantly higher early LAVVR ( *p* < 0.001).

**Fig. 3:**
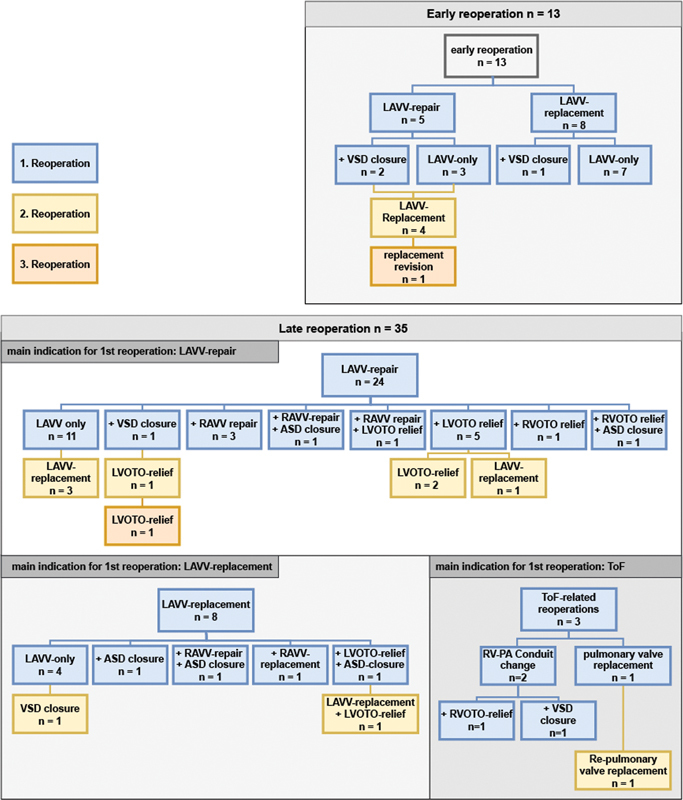
Detailed indications for reoperation stratified by early and late reoperation; ASD, atrial septal defect; LAVV, left atrioventricular valve; LVOTO, left ventricular outflow tract obstruction; PA, pulmonary artery; RAVV, right atrioventricular valve; RV, right ventricle; RVOTO, right ventricular outflow tract obstruction; ToF, tetralogy of Fallot; VSD, ventricular septal defect.

Fig. 3 shows a detailed analysis of indications for reoperations. Twenty-three (36%) procedures had multiple indications. Moderate or higher LAVV insufficiency was the main indication for reoperation with 55/64 (85.9%) procedures, where 45 out of the 48 reoperated patients needed at least one LAVV-related reoperation. At first reoperation, re-repair was possible in 32 (71.1%) patients and valve replacement occurred in 13 cases. Cleft dehiscence was encountered in 9 patients. Seven Patients received a second reoperation for LAVV replacement after re-repair.

Concomitantly to the reintervention of the LAVV in 21/55 procedures, at least one other area was addressed: RAVV re-repair or replacement in 7 cases, residual ASD or VSD closure in 9 cases, RVOTO relief in 2 cases, and LVOTO relief in 6 cases.

Overall, LVOTO relief was performed in 8 (3.2%) patients, of whom 4 required a second relief for recurrence. At the time of the last follow-up, 5 patients showed echocardiographic turbulent flow signs of recurrence of LVOTO not needing reintervention. In 3 (6.25%) patients reoperations related to TOF such as conduit or pulmonary valve replacement were needed.

The need for early and late reoperations were nearly halved over the surgical eras from 9.1 to 4.4% and 21.2 to 12.3%, respectively. Patients undergoing reoperation for LAVVR had significantly reduced long-term survival compared with no reoperation (log-rank *p* < 0.001 as depicted in Fig. 2B ).

While patients with CoA and ToF had significantly higher reoperation rates ( *p* = 0.03 and <0.001, respectively) survival analysis of this subgroup was comparable to the entire cohort. Independent risk factors for reoperation were complex AVSD ( *p* = 0.018) and LAVVR ≥ I–II° after repair ( *p* = 0.002; Table 3 ).

### Infective Endocarditis

During follow-up two (0.8%) patients underwent LAVV replacement for confirmed infective endocarditis. The first patient had confirmed *Streptococcus faecalis* 4.5 months after corrective surgery, whereas in the second patient *Aerococcus urinae* was encountered 18.5 years after correction. Both patients were alive at last follow-up with normal prothesis function.

### Clinical Status at Last Follow-Up

Overall, clinical status of surviving patients ( *n* = 215) at last follow-up was excellent, with 97.2% classified as NYHA (New York Heart Association) I or II, 91.2% having sinus rhythm and 97.7% presenting with good systolic function upon last echocardiography ( Supplementary Material S1 , available in the online version).

Pacemaker implantations were necessary in 22 patients. Early or in-hospital high-grade AV block leading to pacemaker therapy occurred in 13 (5.2%) cases. Further, 9 patients required delayed pacing therapy due to late III° AV-block in 4 and sick sinus syndrome in 5 cases.

## Discussion

Long-term outcome analysis for AVSD correction confirms the marked reduction in perioperative mortality as well as reoperation rate over the last decades. The era effect has been previously reported in studies providing long-term follow-up and is consistent with the findings presented herein. 2 10 11 12 Refinements in diagnostics, surgical technique, and postoperative care, are important contributors in this regard, to state a few. 13 14 Over the surgical eras a noticeable trend toward earlier intracardiac primary correction in case of cAVSD—as opposed to primary PAB—was encountered. Furthermore, inhalative nitric oxide and intensified afterload-lowering medication in the early postoperative period were progressively available.

Surgical repair for cAVSD is recommended between 3 and 6 months of age, to avoid the development of irreversible pulmonary hypertension and progressive changes of AV regurgitation with ensuing congestive heart failure. 3 15 While some centers report excellent outcomes of early repair even in patients before the 3-month threshold, 16 17 several studies show low-weight and younger age to still be associated with complex postoperative course and increased burden of reoperation and mortality. 18 19 Recent studies report the feasibility of very early surgical correction in infants younger than 3 months or weighing less than 3.5 kg. 15 20 However, some studies raise concern regarding promptitude surgery, due to the fragility 18 or anatomical limitations, e.g., annular size. In this cohort, multivariable risk factor analysis identified low weight (<4 kg) and partial cleft closure but not age to be independent predictors of mortality. The decrease in mortality over the surgical eras correlates with the pursuit of complete cleft closure as well as the increased application of additional reconstructive measures such as annuloplasty. Moreover, partial cleft closure was primarily due to dysplastic valve tissue and concerns of significantly reducing orifice area. However, already dysplastic, or damaged valve tissue is prone to further progressive changes due to the continued exposure to the regurgitant volume. 21 Hence, in this study partial cleft closure was associated with a 2.4-fold increase in mortality, due to the ensuing negative sequelae of LAVVR on ventricular dysfunction.

Compared with similar studies, this cohort included a notable proportion (26.4%) of premature patients, again correlating with mortality upon multivariable testing. The subgroup analysis of patients undergoing prior cPAB survival was not significantly decreased after cAVSD correction. Data collected from the current surgical era reveal that cPAB was reserved to only 15 patients. At the last follow-up, all 15 patients were survivors and had good functional status (median follow-up time for this subgroup was 4 years [IQR: 1.7–5.6 years]). Thus, supporting the role of bridging in patients with known additional risk factors associated with mortality. 22

The improved survival warrants the analysis of the postoperative course. Despite markedly improved survival, the need for reoperations and long-term morbidity remains an issue of concern in these patients. 4 23 Thus, a special focus of our study was to evaluate the total burden of reoperations and identify associated risk factors influencing the incidence of repeat surgery as well as survival after repeated corrective surgery.

Most studies report outcomes of surgical reinterventions, with a special focus directed to LAVV pathology. 4 24 25 Conversely, detailed analysis regarding the total burden of reintervention incidence is less often reported. 16 21 While LAVV function remains the cardinal cause for repeat surgery, other indications may play an important role in the clinical development of the affected patients. Previous reports have shown that a significant proportion of corrected patients are susceptible to an increase of LAVVR by at least one grade over follow-up. 26 The indication for repeat intervention is always weighed judiciously, especially in the pediatric population. Prior publications report 34 and 47% of the procedures following AVSD repair, addressing additional lesions. 27 28 This is reflected in the current cohort with 36% of patients undergoing reintervention for multiple indications.

Data analysis reveals that the incidence of repeat surgical corrections is not associated with specific AVSD subtypes. This is in accordance with previous findings by Schleiger et al and Hoohenkerk et al. 11 13 Early reintervention is directly associated with LAVV function as seen in the subgroup analysis, with 3 out of 5 early reinterventions being isolated re-repairs and 7 out of 8 isolated re-replacements. Independent predictors for reoperation were postoperative LAVV regurgitation greater than mild as well as complicated AVSD anatomy. This is in accordance with the predictive value of immediate postoperative LAVV competence with mortality. Hence, underscoring the role of additional measures increases valve competence for durable results. The anatomic features of the valvular tissue and factors associated with complicated AVSD is further reflected in the high incidence of re-replacement in these patients. Other factors contributing to increased volume overload and thus leading to reinterventions were residual ventricular shunts, leading to repeat surgery.

Interestingly, on the other hand, it is the proportion of reinterventions during late follow-up, with 11 out of 24 procedures identified as isolated re-repairs and 4 out of 8 isolated re-replacements. The remaining 3 procedures were associated with Fallot association and primarily addressed the RVOT. Noteworthy, reinterventions due to increased gradient and stenotic development of the reconstructed LAVV remained negligible (2.2%, Supplementary Material S2 , available in the online version).

Postoperative LAVV function is a known predictor of long-term functional status 11 29 30 and in this case survival. Survival after reintervention was significantly reduced (log-rank: *p* < 0.001). This was previously encountered by Pontailler et al and Sojak et al, 24 27 whereas other reports found no difference in mortality in their cohort. 28 While the analysis performed by Ramgren et al 28 showed no significant difference in outcome after repeat surgery, the authors identified the presence of additional cardiac anomalies such as persistent LSVC and previous surgery for CoA to be independent predictors for mortality. The incidence of these anomalies in the present cohort was nearly double the reported incidence in their study. This finding may be responsible for the observed difference in outcome. Additionally, some reports include all operations for AVSD corrections, whereas others exclude patients with associated cardiac anomalies needing surgical correction from their analysis. This in turn complicates the comparison, since the former case reflects survival after reoperations including complex procedures.

Consistent with similar reports, the observed mortality for LAVV replacement at reoperation was higher than LAVV re-repair. 4 24 27 31 This in turn confirms the burden associated with prosthetic solutions, especially in young infants, which is correlated with significant mortality and morbidity. 32

## Limitations

This study is limited by its retrospective character and single-center design. The number of patients in the tAVSD group and in different subgroups was relatively low. This reduces the power of the results and should be interpreted with caution.

Due to the inclusion criteria of this study, patients receiving PAB that did not survive until AVSD repair are not considered. Caution should be exercised when contemplating the results of the patients receiving PAB in this study because of this survivorship bias.

Moreover, due to the long-term character of this study, the risk for information bias should be noted due to changes in diagnostic criteria across the surgical eras, e.g., for pulmonary hypertension.

## Conclusion

Reoperations of the LAVV remain common after AVSD correction. The incidence of reoperation increased in association with early LAVV insufficiency and presence of associated cardiac defects. Surgical and postoperative treatment should focus on early LAVV function. Overall, the pursuit of early repair with focus on LAVV competence and avoidance of pulmonary hypertension improved surgical outcome and survival, since early postoperative LAVV function predicted reoperation. Staged repair is still preserved for patients with significant comorbidities or risk factors associated with mortality until complete repair is feasible. Further analysis of reoperation outcomes is mandatory to guide surgeons and clinicians in treatment and consulting of patients and their primary caregivers.
