# Supplementary material for: A 27-Year Experience with Atrioventricular Septal Defect Correction
Source: Thorac Cardiovasc Surg Rep. 2025 Mar 28;73(Suppl 3):e11–20. doi: 10.1055/a-2536-8640 (PMC11970461; doi:10.1055/a-2536-8640)
Supplement: Supplementary file 1 — Supplementary Material [file 10-1055-a-2536-8640-s1120247359pcc.pdf]

Status of surviving patients at their last follow up	n = 215

Clinical status	
**NYHA-classification**	
NYHA I	189 (87.9%)
NYHA II	20 (9.3%)
NYHA III	5 (2.3%)
NYHA IV	1 (0.5%)
**Cardiac medication**	
Diuretics	19 (8.8%)
ACE-Inhibitors	58 (27.0%)
Digoxin	8 (3.7%)
ß-Blocker	9 (4.2%)
Phenprocoumon	8 (3.7%)
**Pulmonary Hypertension**	8 (3.7%)

ECG	
Sinus rhythm	196 (91.2%)
AV block I°	14 (6.5%)
AV block III°	3 (1.4%)
Ectopic rhythm	8 (3.7%)
Sick-Sinus-Syndrome	3 (1.4%)
Right bundle branch block	
complete	100 (46.5%)
incomplete	35 (16.3%)
**Pacemaker**	
PM-implantation	16 (7.4%)
PM-associated procedures	
1-2 procedures	7 (3.3%)
3-4 procedures	5 (2.3%)

Echocardiography	
residual shunt	24 (11.2%)
LVOTO	11 (5.1%)
**Left ventricle**	
good systolic function	210 (97.7%)
hypertrophy	10 (4.7%)
moderate to severe dilation	4 (1.9%)
**right ventricle**	
hypertrophy	14 (6.5%)
moderate to severe dilation	3 (1.4%)
**left AV-valve regurgitation**	
none-I°	142 (66.0%)
I-II°	62 (28.8%)
>II°	11 (5.1%)
**right AV-valve regurgitation**	
none-I°	193 (89.8%)
I-II°	20 (9.3%)
>II°	2 (0.9%)

Supplementary Material S1 Overview of the clinical status of surviving patients at their last follow-up. ACE, angiotensin-converting enzyme; AV, atrioventricular; LVOTO, left ventricular outflow tract obstruction; NYHA, New York Heart Association; PM, pacemaker.

intraoperative Findings at reoperation n=45	n (%)
dysplastic leaflet tissue	22 (48.9%)
residual cleft	14 (31.1%)
leaflet perforation	11 (24.4%)
repair dehiscence	9 (20.0%)
abnormal leaflet pliability	8 (17.8%)
endocarditis	5 (11.1%)
stenosis after prior repair	1 (2.2%)

Supplementary Material S2 Intraoperative findings at reoperation.

 © 2025. The Author(s).
